# Machine Learning Techniques Applied to COVID-19 Prediction: A Systematic Literature Review

**DOI:** 10.3390/bioengineering12050514

**Published:** 2025-05-13

**Authors:** Yunyun Cheng, Rong Cheng, Ting Xu, Xiuhui Tan, Yanping Bai

**Affiliations:** 1 School of Information and Communication Engineering, North University of China, Taiyuan 030051, China; s1808004@st.nuc.edu.cn; 2 School of Mathematics, North University of China, Taiyuan 030051, China; chengro@nuc.edu.cn (R.C.); xuting@nuc.edu.cn (T.X.); tanxiuhui@nuc.edu.cn (X.T.)

**Keywords:** COVID-19, public health, predictive modelling, machine learning, artificial intelligence

## Abstract

COVID-19 was one of the most serious global public health emergencies in recent years, and its extremely fast spreading speed had a profound negative impact on society. A comprehensive analysis and prediction of COVID-19 could lay a theoretical foundation for monitoring and early warning systems. Since the outbreak of COVID-19, there has been an influx of research on predictive modelling, with artificial intelligence (AI) techniques, particularly machine learning (ML) methods, becoming the dominant research direction due to their superior capability in processing multidimensional datasets and capturing complex nonlinear transmission patterns. We systematically reviewed COVID-19 ML prediction models developed under the background of the epidemic using the PRISMA method. We used the selected keywords to screen the relevant literature of COVID-19 prediction using ML technology from 2020 to 2023 in the Web of Science, Springer and Elsevier databases. Based on predetermined inclusion and exclusion criteria, 136 eligible studies were ultimately selected from 5731 preliminarily screened publications, and the datasets, data preprocessing, ML models, and evaluation metrics used in these studies were assessed. By establishing a multi-level classification framework that included traditional statistical models (such as ARIMA), ML models (such as SVM), deep learning (DL) models (such as CNN, LSTM), ensemble learning methods (such as AdaBoost), and hybrid models (such as the fusion architecture of intelligent optimization algorithms and neural networks), it revealed that the hybrid modelling strategy effectively improved the prediction accuracy of the model through feature combination optimization and model cascade integration. In addition, we compared the performance of ML models with other models in the COVID-19 prediction task. The results showed that the propagation of COVID-19 is affected by multiple factors, including meteorological and socio-economic conditions. Compared to traditional methods, ML methods demonstrated significant advantages in COVID-19 prediction, especially hybrid modelling strategies, which showed great potential in optimizing accuracy. However, these techniques face challenges and limitations despite their strong performance. By reviewing existing research on COVID-19 prediction, this study provided systematic theoretical support for AI applications in infectious disease prediction and promoted technological innovation in public health.

## 1. Introduction

With the advancement of science and technology, healthcare technology and related facilities are constantly improving. However, it is undeniable that new epidemics of infectious diseases continue to break out from time to time across the globe, and it is estimated that about 30 percent of global deaths each year are caused by infectious diseases. Major infectious diseases that have occurred globally since 2000 include SARS [1], H1N1 [2], and Ebola [3]. The spread of these infectious diseases has triggered social panic on the one hand, seriously affecting the stability and development of society, and poses a serious threat to the safety of human life on the other hand [4], as shown in Table 1. Notably, COVID-19 has rapidly spread across the world since the first case was reported in December 2019, bringing a far-reaching global impact. The World Health Organization (WHO) declared COVID-19 to be a global pandemic in March 2020 [5], and with the exponential growth of cases, it has become one of the most destructive global epidemics in modern times.

The spread of COVID-19 has not only led to the loss of a large number of lives, especially the higher mortality rate of the elderly and patients with underlying conditions [6], but also has a long-term impact on global medical resources, economic operation and social order. However, with the variation of the COVID-19 virus and the strengthening of control measures, its harmfulness has gradually decreased. In May 2023, the WHO announced the official end of the COVID-19 pandemic, and the world gradually entered a new normal of coexistence with viruses. However, as the “main culprit” that has affected us for several years, it is necessary to learn lessons from the spread of COVID-19 and conduct in-depth research, which will be important for our future response to the prevention and control of similar infectious diseases.

**Figure 1:**
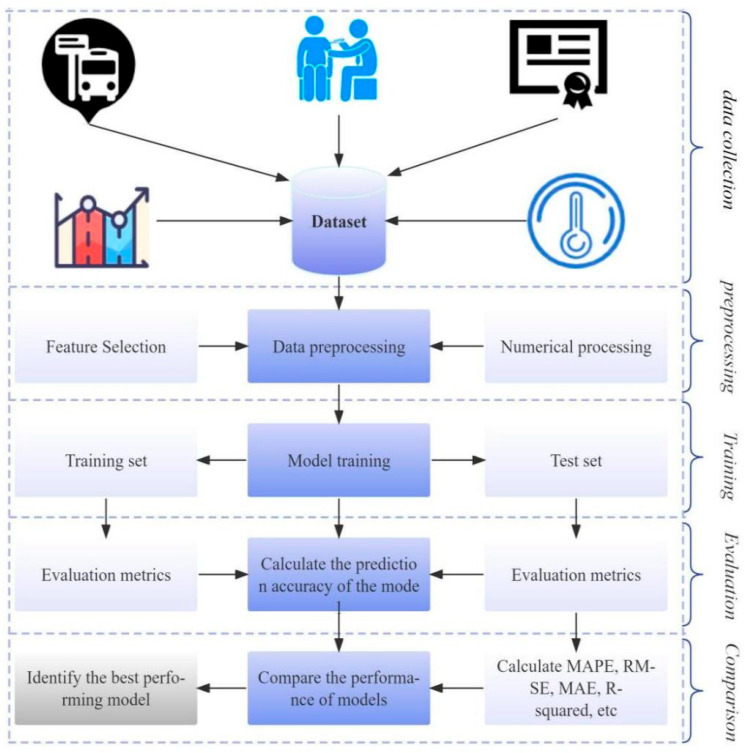
The process of ML prediction.

As the first infectious disease to fully erupt in the digital age, the prevention and control of COVID-19 highlights the limitations of traditional methods. Although the epidemic has largely subsided, its remaining scientific problems urgently need to be solved, such as how to extract transmission patterns from massive heterogeneous medical data, and how to balance model interpretability and prediction accuracy. Existing research indicates that machine learning (ML) and deep learning (DL) techniques have shown potential in predicting disease progression in COVID-19 patients [7], analyzing epidemic transmission trends [8], and optimizing medical resource allocation [9] by mining multimodal data such as electronic health records (EHR), movement trajectories, and social media. Compared to traditional statistical models, the core advantage of ML and DL techniques lies in their ability to handle high-dimensional nonlinear relationships. For example, long short-term memory networks (LSTM) can capture the long-term dependencies of COVID-19 epidemiological curves. More importantly, ML models can adapt to dynamic changes in pathogens through continuous learning, such as during the Delta and Omicron variants, the model based on optimization algorithms can quickly update parameters without the need for complete reconstruction. This characteristic makes it a key tool for predicting infectious diseases under the “new normal”. As shown in Figure 1, ML plays an important role in the prediction of COVID-19.

However, the current application of ML in COVID-19 presented a fragmented feature: multiple COVID-19-related studies were scattered across tasks such as diagnosis, detection, and classification. Aslani and Jacob reviewed 30 papers using 2D/3D deep convolutional neural networks (CNN) combined with transfer learning to detect COVID-19, explored how the DL approach can detect COVID-19, and highlighted several limitations of the proposed approach [10]. Chen et al. summarized the latest development of COVID-19 multi-modal ML, and the consideration of model evaluation in future research. The multi-modal COVID-19 data investigated in the literature are summarized, including symptomatic and other clinical data, laboratory tests, imaging, pathology, physiology and other histological data [11].

Sailunaz et al. analyzed several COVID-19 image analysis methods, surveyed contributions of existing research, available image datasets, and performance metrics in recent work. They also discussed challenges and future research scope in the fight against the COVID-19 outbreak from an artificial intelligence perspective [12]. Habashi reviewed a number of emerging AI-based methods for diagnosing COVID-19 using routine blood tests. The review included 92 studies, and the authors identified the models, datasets, and performance metrics in each study [13]. Das et al. explored available ML and DL models for COVID-19 detection, including 50 articles. They categorized these methods into ML, DL, and combined ML+DL groups. They concluded that both ML and DL can classify COVID-19 and non-COVID-19 from X-ray and CT images with over 99% accuracy [14]. Soda et al. combined chest X-ray images, clinical data and artificial intelligence methods to identify individuals at severe risk in patients with COVID-19 [15]. Prinzi et al. developed an interpretable ML model based on clinical, laboratory and radiological characteristics to predict the prognosis of patients with COVID-19 [16]. Wu et al. developed a non-invasive and easy to use prognostic tool to predict the adverse outcome of COVID-19 patients through chest CT images and radiomics models, combined with the least absolute contraction and selection operator (LASSO) and fine grey competitive risk regression [17]. Wang et al. established a radiation omics model, a clinical model and a combined model to predict the disease progression of COVID-19 patients [18]. Signoroni et al. designed an end-to-end deep learning architecture, which successfully predicted the degree of lung damage in patients with COVID-19 through weak supervised learning strategies and the Brixia scoring system [19].

However, few studies have systematically addressed the following methodological questions: (1) What is the impact of different data types on the performance of COVID-19 prediction models? (2) How should we handle errors or inaccurate information that occur during data collection? (3) What is the performance of traditional prediction models and ML models in predicting the spread of COVID-19? (4) How can we establish a COVID-19 hybrid prediction model by combining multiple algorithms? To address these gaps, we conducted a systematic review of the research on ML-driven COVID-19 transmission prediction from 2020 to 2023 for the first time. Through a multi-level classification framework and analysis of six hybrid modelling strategies, this review revealed both the advantages and limitations of current methodologies. Significantly, we proposed a “data-algorithm-evaluation” adaptation framework, which provides theoretical foundations for developing intelligent early warning systems for infectious diseases.

## 2. Methods

This review focused on studies related to the use of ML methods to predict the COVID-19 pandemic between 2020 and 2023. Following the Preferred Reporting Items for Systematic Reviews and Meta-Analyses (PRISMA) guidelines [20], we implemented a three-stage systematic review process: defining the research question, developing a search strategy to select the literature, and finally extracting and analyzing the relevant content. Table 2 presents the research questions defined in this review. Additionally, we identified the attributes required to answer research questions and designed a table for each selected major study, as shown in Appendix A Table A2.

### 2.1. Search Strategy

Three databases—Web of Science, Springer, and Elsevier—were searched for relevant studies. Table 3 summarizes the search queries used in each database. First, a relevant topic was identified: “Predicting the COVID-19 pandemic using machine learning”. This topic was then divided into three keywords: “Machine learning”, “Prediction”, and “COVID-19”. These keywords were then used to construct various queries for each research database, based on their respective syntax.

### 2.2. Eligibility Criteria

Reasonable screening criteria were developed to identify studies directly relevant to the topic of this review. We used both the following inclusion and exclusion criteria in the screening process:

Inclusion criteria:

(1) Used at least one ML technique to predict COVID-19 transmission trends.
(2) Page length 8+.
(3) Reported predictive performance metrics of ML models.
(4) Experimentation on different datasets related to COVID-19.
(5) Limited to journal articles.

Exclusion criteria:

(1) Full text unavailable.
(2) Not relevant to COVID-19 prevalence or trend projections.
(3) No practical theoretical research (e.g., survey and review papers).

### 2.3. Study Selection

**Figure 2:**
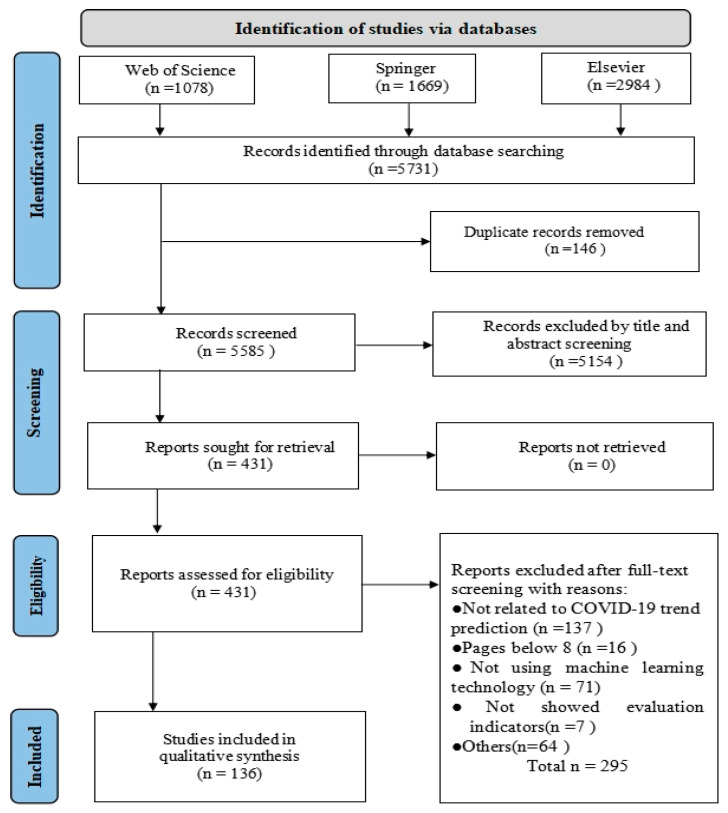
The flowchart of the search process.

Figure 2 shows a flowchart of the search process. First, we defined the list of search terms in the search query. Then, the titles and abstracts of the articles were read to exclude any studies that did not involve ML to predict the COVID-19 pandemic, according to the established inclusion and exclusion criteria. Finally, the full article was read, and additional studies were removed based on the exclusion criteria.

### 2.4. Study Risk of Bias Assessment

Initiating quality assessment poses an initial challenge due to lack of a universally accepted definition of “quality” in research. In response, we devised a comprehensive questionnaire to evaluate relevance and robustness of major research efforts. Table 4 enumerates key aspects of quality assessment, encompassing considerations like sample size, data availability, handling missing data, model comparisons, reporting performance indicators, and thorough exploration of limitations in related studies.

The 10 assessment questions (AQs) were rated as follows: studies meeting the criteria received a score of 2, those meeting the criteria moderately were assigned a score of 1, and studies not meeting the criteria received a score of 0. Studies achieving a total score equal to or above 17 were categorized as of very high quality, those with scores ranging from 14 to 16 were deemed high quality, and studies scoring between 11 and 13 were classified as moderate quality. Studies scoring below 10 were considered low-quality. Two authors (Y.C. & Y.B.) independently conducted the quality assessment of the included studies, resolving any discrepancies through discussion and consensus. In cases where consensus could not be reached, a third author (R.C.) was consulted to make the final decision.

In addition, there may be publication bias in the literature screening stage, such as studies with positive results are more likely to be published. Second, due to team resource constraints, only English literature was included in this review, which may have omitted research results from non-English-speaking regions. To address the risk of subjectivity in the data extraction process, we implemented a standardized process of two-person independent screening and extraction: two researchers performed title/abstract screening, full-text assessment, and data extraction, respectively, and disagreements were resolved through arbitration by a third researcher.

### 2.5. Data Synthesis

The purpose of data synthesis is to analyze and summarize information from selected articles to obtain conclusive answers to research questions. One piece of evidence may have limited evidential strength, but aggregating many pieces of evidence can make a point stronger. We explored multiple ML methods for predicting, encompassing diverse approaches to feature sub-selection and utilizing various datasets. Additionally, a comprehensive examination and evaluation of quantitative data were conducted, focusing on performance indicators such as mean absolute percentage error (MAPE), root mean square error (RMSE), and mean absolute error (MAE). Subsequently, the data gathered from the primary study underwent synthesis employing a range of techniques. To address the research questions, we employed visualization techniques, including line graphs, box plots, pie charts, and bar charts. Furthermore, we utilized tables to succinctly summarize and present the results.

## 3. Results

A total of 5731 studies were retrieved from digital databases, of which 146 duplicates were excluded and 5154 were removed because their titles and abstracts did not meet the inclusion\exclusion criteria. Thereafter, 431 studies were eligible for full-text analysis through the screening process. After reviewing the full text, 295 of these were excluded. Ultimately, 136 primary studies were included in this systematic review.

### 3.1. Quality Assessment

For quality assessment, our aim was not to exclude studies based on measured quality, but rather to evaluate the overall quality of the published research and identify potential methodological strengths or weaknesses. The quality scores in this study were strictly derived from 10 predefined evaluation criteria (Table 4), encompassing data integrity, methodological description, reproducibility, and other dimensions, with no association with specific algorithmic performance or baseline task selection. This scoring system was designed to preserve independence between methodological rigor and research content. Consequently, instead of presenting granular quality scores in the main text, we demonstrate the distribution of studies across quality assessment domains and summarize key findings in Table 5. Approximately 70% (95) of included studies exhibited high or very high quality, while only 4% (5) were classified as low-quality. A comprehensive breakdown of quality assessments is provided in Appendix A Table A1.

### 3.2. Bibliometric Analysis

**Figure 3:**
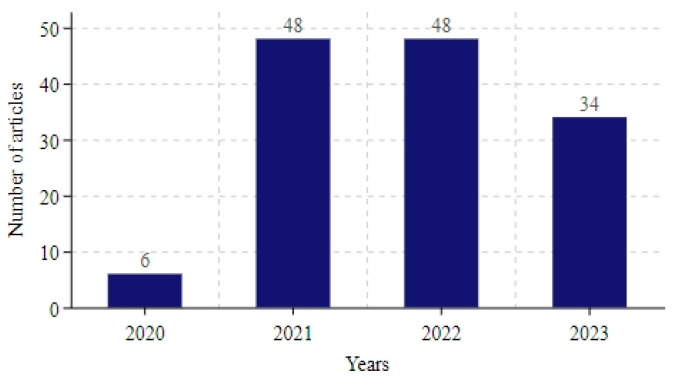
Number of articles included each year.

Figure 3 illustrates the distribution of the number of articles per year. At the beginning of the 2020 outbreak, there were relatively few studies on COVID-19 projections. Between 2021 and 2022, the number of studies increased significantly and reached a peak. By 2023, the number of research articles had gradually decreased. This trend reflects the diminishing impact of the COVID-19 virus on humans, leading to a gradual decline in research on specific topics and a possible shift in the direction of research to other areas.

**Figure 4:**
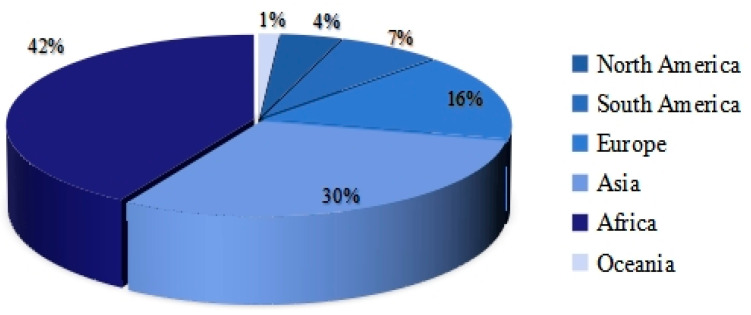
Distribution of research areas by country.

Of the 136 studies involved, we analyzed in detail the distribution of study areas in the articles, as depicted in Figure 4. Researchers delved into COVID-19 transmission patterns across 70 countries spanning diverse continents. Specifically, 29 studies focused on Africa (including Nigeria, South Africa, Egypt, etc.), 21 on Asia (including India, China, Bangladesh, etc.), 11 on Europe (including Italy, Spain, the UK, etc.), 5 on South America (including Brazil, Argentina, etc.), and 3 on North America (including the USA, Mexico, and Canada). Additionally, a singular study pertained to Australia in Oceania. Notably, the Antarctic region was excluded from the study focus.

### 3.3. Basic Content of COVID-19 Prediction

#### 3.3.1. Dataset

Multiple datasets were used in the COVID-19 prediction study, contributing to a comprehensive understanding of the epidemic situation, assessment of the effectiveness of interventions, and development of future response strategies. The following are the main datasets used in the literature:

**COVID-19 Dataset:** This dataset included confirmed, death, and recovered cases reported on a daily basis globally and by country/region.

**Meteorological Data:** This dataset encompassed a wide array of parameters, including average humidity, maximum and minimum temperature, maximum and minimum relative humidity, precipitation, surface down welling solar radiation, wind speed, and air quality indicators (e.g., O_3_, PM_10_, PM_2.5_, SO_2_, NO_X_, NO_2_, CO, etc.). It was utilized by 19 studies, obtained from various primary sources.

**Vaccine dataset:** It mainly covered COVID-19 vaccination status and contained the following indicators: people fully vaccinated (all doses), number of people vaccinated (at least one dose), total vaccinations, among others. Seven studies utilized this dataset.

**Mobility dataset:** This dataset covered mobility trends and urban activity parameters, such as city migration and emigration indices, intracity travel intensity, intercity traffic flow, and changes in footfall at locations like parks, pharmacies, and bus stops.

**Restriction Dataset:** This dataset included restrictions imposed by governments during the epidemic to slow the spread of the virus, such as international travel controls, cancellation of public events, closure of public transport, stay-at-home requirements, and restrictions on gatherings.

In addition, other datasets were utilized to analyze the outbreak’s multifaceted impacts, such as demographic data, economic indicators, and social media or internet search trends.

#### 3.3.2. Data Preprocessing

During the actual collection of COVID-19 case data, missing values and outliers were common phenomena. These issues stemmed primarily from factors such as errors in data entry, sample bias, and the inherent complexity of the pandemic. Inaccurate or incomplete data could severely compromise the training of prediction models, preventing them from accurately capturing and fitting time-series trends. Therefore, adequate preprocessing of COVID-19 data was critical.

**Figure 5:**
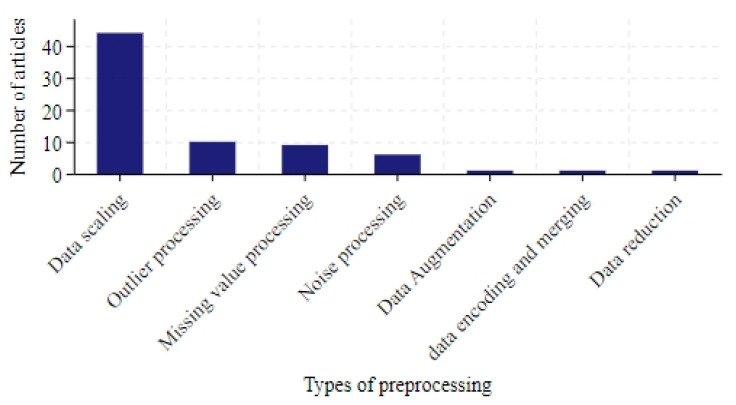
The number of articles in data preprocessing.

Among the 136 studies analyzed, only 67 (50%) described their data preprocessing methods. Figure 5 illustrates the number of different preprocessing techniques used in these studies. The methods are categorized as follows:

**Data scaling:** In total, 44 studies applied scaling techniques, with normalization [21] and standardization [22] being the most common.

**Outlier processing:** The most straightforward involved directly removing data points containing outliers [23]. This could be achieved by setting thresholds or using detection algorithms such as K-means [24]. However, excessive removal risked losing valuable information. An alternative was to replace outliers with substitutes like the median, mean, or interpolation-based estimates [25].

**Missing value processing:** Interpolation was widely used to estimate missing values. Statistical approaches (e.g., mean, median [26,27]) or linear/polynomial interpolation [28,29,30] were common.

**Noise processing:** Techniques aimed to reduce noise and enhance trend identification: Ref. [31] smoothed the time series by calculating the average of the data points over a sliding window. Ref. [32] used singular spectrum analysis to decompose the time series into a number of components, including trend, periodicity and noise, and dealt with the noise by analyzing these components. Ref. [33] used wavelet transform to decompose the time series into wavelet components of different scales and frequencies and smoothed the data by removing the high frequency noise. Ref. [34] tested the smoothness of the time series by using the Dickey–Fuller test and removed the seasonal trend from the non-smooth data set.

**Others:** Additional approaches included data encoding, merging, reduction, and augmentation.

#### 3.3.3. Evaluation Indicator

Table 6 lists the different types of performance metrics used in the selected studies. RMSE was the most commonly evaluated metric, appearing in 93 studies. Following closely were MAE (62 studies), MAPE (50 studies), and R-squared (47 studies). Additionally, mean squared error (MSE) and accuracy stood out as other frequently utilized metrics. To assess reproducibility, we introduced two key metrics: code openness and data availability. The results showed that despite significant progress in prediction accuracy, only 16.9% of the studies provided open code, and 50.7% offered publicly accessible datasets. This phenomenon was particularly prominent in machine learning studies, which demonstrated significantly lower average reproducibility compared to traditional methods.

### 3.4. Machine Learning Models for COVID-19 Prediction

**Figure 6:**
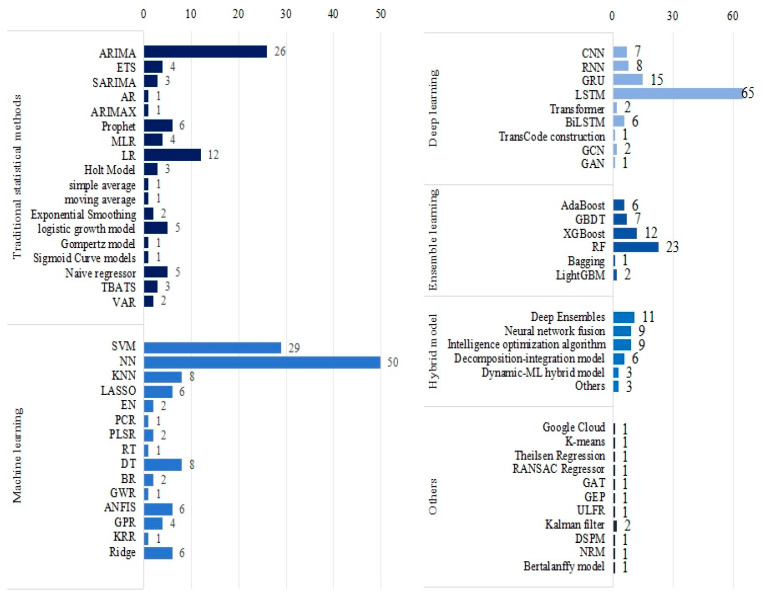
The ML methods used in the articles.

Various ML techniques were applied in the included studies, with Figure 6 providing a comprehensive overview of the methods used. Neural networks (NN) and support vector machines (SVM) emerged as the most commonly employed ML models in the literature, while long short-term memory (LSTM) was the predominant deep learning model for COVID-19 trend prediction, appearing in 64 studies. Additionally, the auto regressive integrated moving average (ARIMA), random forest (RF), gated recurrent unit (GRU), linear regression (LR), and XGBoost were widely utilized. Rashed and Hirata employed LSTM and Google Cloud technology, integrating meteorological and mobility data to forecast the number of disease cases [35]. Didi et al. considered tweets with external features and vaccine-related data, applying multiple ML models such as LSTM, prophet, and SVR to predict confirmed and death cases [36]. Yu et al. utilized ARIMA, feed forward neural network (FNN), multi-layer perceptron (MLP) neural network, and LSTM to predict COVID-19 cases within a 14-day window [37]. Kavouras et al. tested four different DL methods, namely Conv1D-LSTM, GRU, LSTM, and recurrent neural networks (RNN), forecasting daily data on cases, deaths, hospitalizations, and daily admissions to Intensive Care Units (ICU) [38]. Yeung et al. employed various ML models, including ridge regression, decision tree (DT), RF, AdaBoost Regression, and SVR, to predict the growth of COVID-19 infection cases [39].

**Figure 7:**
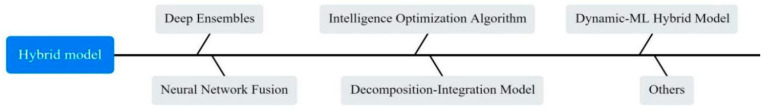
The classification of hybrid models used in the articles.

However, no single model can perform best in all situations. Different algorithms may show better performance on different subsets of data or different aspects of the problem. As a result, researchers are increasingly favouring the construction of more powerful predictive models by combining multiple approaches. As shown in Figure 7, we categorized the predictive models in different hybrid ways.

#### 3.4.1. Meta-Heuristic Algorithmic Optimization Models

**Figure 8:**
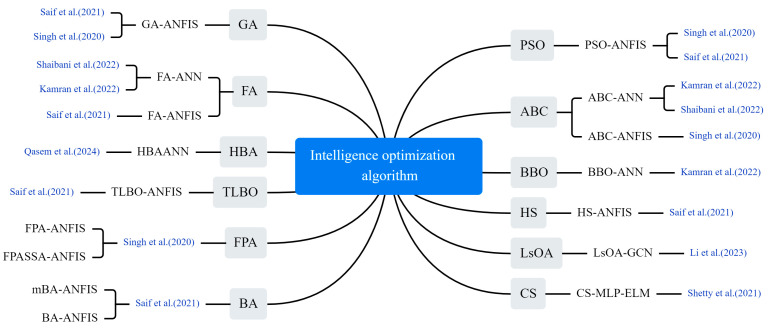
The optimization algorithm used in the selected article [7,40,41,42,43,44,45].

Hyper-parameter optimization of neural networks has long been a hotly studied issue among scholars. To improve the accuracy of epidemic trend prediction, swarm intelligence optimization algorithms are widely used to optimize the parameters of machine learning (ML) models. These algorithms do not rely on the specific structure of the problem but explore the solution space by simulating the natural behavior of organisms, and they have become an important tool for enhancing model prediction performance. As shown in Figure 8, a series of optimization algorithms were applied to COVID-19 prediction, including a genetic algorithm (GA), particle swarm optimization (PSO), differential evolution (DE), firefly algorithm (FA), harmony search (HS), teaching–learning-based optimization (TLBO), bees algorithm (BA), mutation-based bees algorithm (mBA), lioness optimization algorithm (LsOA), honey badger algorithm (HBA), artificial bee colony (ABC), cuckoo search (CS) algorithm, biogeography-based optimization (BBO), improved beetle antennae search algorithm (IBAS), and sparrow search algorithm (SSA).

Saif et al. modified the standard BA by introducing a mutation process and used a hybrid model (mBA-ANFIS) comprising mBA and an adaptive neuro-fuzzy inference system (ANFIS) to predict the number of diagnosed cases. They compared the proposed model with the standard ANFIS model and several other models, including GA-ANFIS, DE-ANFIS, HS-ANFIS, TLBO-ANFIS, ANFIS, FA-ANFIS, PSO-ANFIS and BA-ANFIS [40]. Li et al. proposed a graph convolutional network (GCN) prediction model based on LsOA. The feature matrix was first filtered using LsOA. Then, a GCN was used to obtain the spatial features of the epidemiological related data and generate the prediction results [41]. Shaibani et al. predicted new cases of COVID-19 using a hybrid artificial neural network (ANN) in which the ANN combines the ABC and the FA and selects the optimal model with the highest accuracy [42]. Qasem built a hybrid intelligence model, HBA-ANN, by hybridizing ANNs with HBA and compared it with standalone neural networks and gene expression programming (GEP) models [43]. Shetty and Pai utilized the CS algorithm to select parameters for an extreme learning machine (ELM) prediction model. They compared the model’s performance with a conventional model that used an auto correlation function to select parameters [44]. Singh et al. used RF and Kalman filter to analyze the spread of COVID-19, and com-pared the results with various models such as PSO-ANFIS, ABC-ANFIS, FPA-ANFIS and FPASA-ANFIS [45].

#### 3.4.2. Deep Ensembles Models

**Figure 9:**
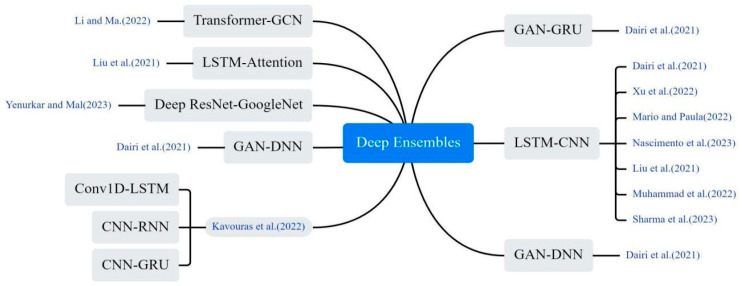
The deep ensembles method used in the selected article [23,29,31,38,47,48,49,50,51,52].

The CNN-LSTM hybrid model stands out as the most widely adopted approach, as illustrated in Figure 9. The CNN extracts spatiotemporal features from training data through convolutional and pooling operations, generating hierarchical representations. This CNN-generated embedding serves as input to the LSTM network for predicting COVID-19 case. The synergistic architecture not only efficiently extracts and transforms multimodal features but also captures complex temporal dependencies. This dual functionality significantly enhances the accuracy of COVID-19 prediction models [29,46,47,48,49]. Li and Ma proposed a hybrid model integrating an enhanced Transformer with a GCN for COVID-19 prediction. This model stands out for its unique ability to extract comprehensive time series information using a multi-attention mechanism, followed by further correlation aggregation through GCN [23]. Liu et al. enhanced the LSTM model by incorporating a multi-attention mechanism, enabling the model to focus on crucial segments of the time series and avoid biased values [50]. Yenurkar et al. created a hybridization by combining the ResNet and GoogleNet models, amalgamating these two DL processes for the predictive task [51]. Dairi et al. used a variety of ML methods to predict the confirmed and rehabilitated cases of COVID-19, and the results showed that LSTM-CNN model performed best [52]. 

#### 3.4.3. Neural Network Fusion Models

**Figure 10:**
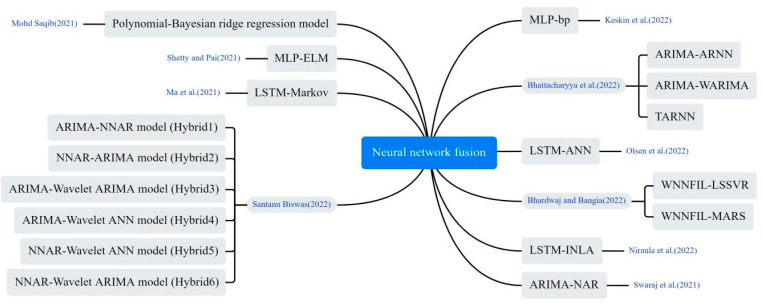
The neural network fusion method used in the selected article [28,33,44,53,54,55,56,57,58,59].

The researchers constructed more comprehensive prediction models by combining multiple neural networks in different ways, including stacking and concatenation. As shown in Figure 10, Olsen et al. developed a DL hybrid model LSTM-ANN, in which LSTM captures the temporal information of the input sequence, and MLP maps it to a higher dimensional representation to extract key features from the input sequence [28]. Saqib hybridized Bayesian ridge regression with polynomials of degree *n* and used probability distributions to estimate COVID-19 confirmed and deceased cases [33]. Ma et al. proposed an innovative LSTM Markov model. It constructs the probability transition matrix of the Markov model based on the prediction error of the LSTM model. Then, the output data of the LSTM model is combined with the prediction error of the Markov model to obtain the final prediction result [53]. Bhardwaj and Bangia proposed a wavelet neural network (WNN) model for predicting the trend of COVID-19. The model combined time series and local frequency decomposition of discrete waveform signals, and was trained using both least squares support vector machine (LSSVR) and multivariate adaptive regression spline (MARS) methods [54]. Niraula et al. proposed a Bayesian LSTM approach. The LSTM is first used to predict the number of future cases and then the output predictions are embedded as expected in a Bayesian Poisson regression model [55]. Bhattacharyya et al. proposed a hybrid TARNN model based on the Theta method and ARNN model, comparing it with traditional individual models. The proposed TARNN model outperforms all traditional individual and hybrid models [56]. Keskin et al. used the MLP structure and experimentally compared different network topologies, concluding that the Levenberg–Marquardt Back Propagation (LMBP) algorithm performed best among the feed-forward back propagation algorithms [57]. Santanu combined wavelet decomposition with ARIMA and NNAR models, developing six residual mixture models. Initially, the original time series was predicted using these models; subsequently, the residuals of the predictions underwent further modelling [58]. Swaraj et al. proposed an integrated model, combining ARIMA and Nonlinear Auto Regressive Neural Network (NAR). ARIMA extracted the linear correlation, while NAR modeled the ARIMA residuals, containing the data’s nonlinear components [59].

#### 3.4.4. Decomposition–Integration Models

**Figure 11:**
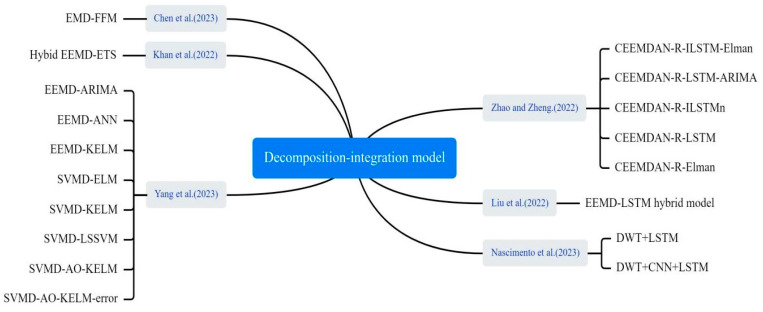
The decomposition–integration method used in the selected article [48,60,61,62,63,64].

Decomposition–integration architecture, as an effective hybrid modelling approach, demonstrates its great potential in infectious disease prediction, as shown in Figure 11. Zhao and Zheng developed an integrated prediction model. First, complete ensemble empirical mode decomposition (CEEMD) was applied to decompose the data. Then, the decomposed empirical mode component (imf) was reconstructed into four sequences representing high, medium, low, and trend terms by calculating alignment entropy and averaging period. Finally, each component sequence was predicted using ILSTM and Elman algorithms. The results were combined to yield the final predictions [60]. Liu et al. proposed a hybrid model based on integrated empirical pattern decomposition (EEMD) and LSTM to predict the trend of COVID-19 [61]. Yang et al. proposed a SVMD-AO-KELM-error method for short-term COVID-19 case prediction. They used a SSA to improve the variational mode decomposition (VMD) of COVID-19 case data. Then, an Adaptive Optimization-based Kernel Extreme Learning Machine (AO-KELM) was used to predict the Intrinsic Mode Function (imf) components and residuals. Finally, they reconstructed the prediction results and error prediction results for each component [62]. Khan et al. introduced a novel hybrid model, integrating empirical mode decomposition and error trend seasonality (EEMD-ETS), designed to predict the COVID-19 pandemic. The model addresses challenges of data variability and complexity inherent in pandemic data [63]. Chen et al. smoothed complex, variable epidemic data via EMD, then trained the trend at varying time scales using ELM to obtain predicted values. Finally, they used ANFIS to fit and generate epidemic prediction results [64].

#### 3.4.5. Dynamic–ML Hybrid Model

**Figure 12:**
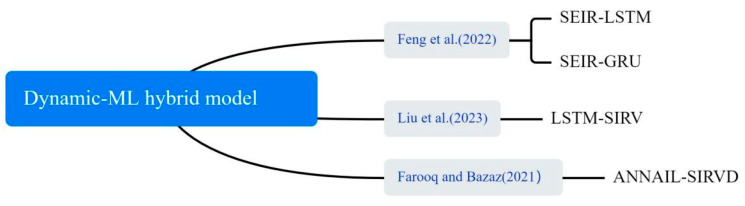
The dynamic–ML hybrid method used in the selected article [65,66,67].

In the application of COVID-19 outbreak prediction, hybrid dynamics models have also been shown to improve the accuracy of predictions, as shown in Figure 12. ML was used to fit and optimize parameters in the dynamics model, such as infection rates and transmission rates, so that the model could more accurately reflect the complex transmission patterns of the outbreak. Liu et al. used NAR, LSTM, ARIMA, Gaussian and polynomial functions to predict the transmission rates β. The predicted transmission rates $\beta_{s}$ were then incorporated into the SIRV model to predict the number of confirmed cases of COVID-19 [65]. Feng et al. described a SEIR-LSTM/GRU algorithm with time-varying parameters for predicting confirmed and recovered cases in the United States. The SEIR model parameters were estimated from training data and used as time-dependent inputs to train the LSTM and GRU models. The resulting SEIR model parameters were applied for data fitting and prediction [66]. Farooq and Bazaz proposed an adaptive incremental learning technique based on ANNs for online learning of SIRVD model parameters [67].

#### 3.4.6. Other Models

**Figure 13:**
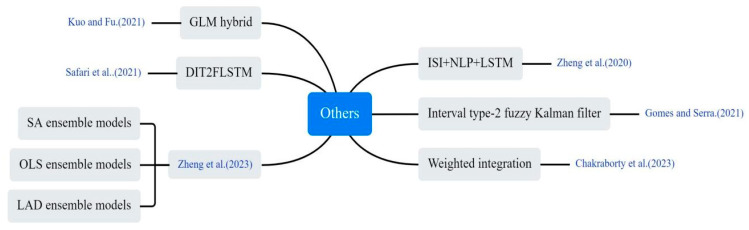
Other innovative methods used in the selected article [25,26,32,68,69,70].

Some non-traditional and innovative approaches were applied to COVID-19 prediction, as shown in Figure 13. Zheng et al. utilized natural language processing (NLP) techniques to extract semantic features from news reports concerning outbreak prevention measures and public sentiments. These features were integrated into an LSTM network, enabling prediction of infected individuals by adjusting infection rate within a conventional infectious disease model [25]. Kuo et al. utilized a hybrid technique derived from the general linear model (GLM) to integrate outcomes from eight base models. By weight-adjusting the combined results, they enhanced the hybrid model’s performance [26]. Gomes and Serra combined ML, fuzzy logic, and clustering algorithms to propose a computational model for adaptively tracking and predicting the dynamic propagation of COVID-19. The model was tested on an experimental dataset from Brazil [32]. Zheng et al. utilized three decision-tree-based ML algorithms (RF, XGBoost, and LightGBM) to predict new daily cases. They also applied three linear integration methods (simple average, least squares, and least absolute deviation) to enhance prediction accuracy [68]. Chakraborty et al. employed a cross-country pre-training strategy utilizing COVID-19 data from USA, Brazil, Spain and Bangladesh. The prediction performance for the Indian outbreak was improved by using GRU and weighted average methods [69]. Safari et al. proposed a new Deep Interval Type-2 Fuzzy LSTM (DIT2FLSTM) model for predicting the incidence of COVID-19, which improved the accuracy and stability of the prediction by introducing the interval type-2 fuzzy set theory [70].

### 3.5. Performance Comparison Between Machine Learning Models and Other Models

We conducted a comparative analysis between the performance metrics of ML models and those of non-ML models. To make the comparison results more reliable, we discussed the performance of both models in the same article. Overall, 36 articles employed both ML and non-ML models. Table 7 provides a comparative assessment of their performance. Remarkably, ML models outperformed non-ML models in over 86% of studies, while non-ML models surpassed ML models in only 14% of cases examined.

We specifically analyzed the results of the comparison with non-ML techniques in at least two datasets. Kumar et al. used various ML, time series, and DL models to predict confirmed, deceased, and recovered cases of COVID-19 in ten different countries, and RF and Stacked LSTM performed the best [21]. Silva et al. used ML and LR to make spatio-temporal predictions of case and death distribution in Brazil and each federated unit, with linear regression giving best predictions for Pernambuco and all of Brazil [71]. Gao et al. proposed a spatio-temporal attention network (STAN) for pandemic prediction, outperforming traditional epidemiological models like SIR and SEIR in both long- and short-term prediction, achieving up to 87% reduction in mean square error [72]. Malki et al. compared the proposed DT model with various state-of-the-art models (RF and ARIMA) and the DT model has better performance [73]. Chaurasia and Pal implemented several forecasting techniques: naive method, simple average, moving average, single exponential smoothing, holt linear trend method, Holt–Winters method and ARIMA. The naive approach was found to be the most applicable [74]. Khakharia et al. made predictions for 10 densely populated countries using nine ML models, including ARIMA, ARMA, SVR, LR, XGBoost, BRR, Holt–Winters, and RF. The ARIMA model performed well in predicting COVID-19 epidemic development, exhibiting high predictive effectiveness compared to other models [75]. Rahman and Chowdhury compared the predictive accuracy of ARIMAX and XGBoost methods for accurate modelling of COVID-19 incidence, with the ML-based XGBoost model outperforming the ARIMAX model in predicting COVID-19 incidence in SAARC countries [76].

## 4. Conclusions

The study of infectious diseases not only relies on traditional public health and medical methods, but also requires interdisciplinary integration with the help of technologies from computer science, artificial intelligence and other fields. Combining technologies from these different fields, especially the latest ML techniques, is an important challenge for the future. In this study, a systematic review of ML methods for COVID-19 prediction was conducted through the PRISMA approach. In this study, we systematically reviewed ML methods for COVID-19 prediction through the PRISMA method. We first analyzed the basic content of COVID-19 prediction, including key factors such as dataset, data preprocessing, and evaluation indicators. Secondly, the application of various ML methods in COVID-19 prediction was explored, including classic supervised learning methods, unsupervised learning methods, and deep learning techniques, and further detailed classification of hybrid models was conducted. Finally, the performance of ML models in predicting COVID-19 was compared with other traditional prediction models such as epidemiological models and statistical models. The key findings of this review are summarized below:

- The spread of infectious diseases is influenced by a variety of factors, including historical cases, meteorological conditions, and socio-economic factors such as population movements. Consideration of these influences in COVID-19 projections helps to more fully understand and predict trends and impacts of outbreak spread.
- Data scaling, outlier processing, missing value processing and noise processing are commonly used in data preprocessing methods.
- LSTM and SVM are the most commonly used ML models. The prediction accuracy of the model can be effectively improved by various hybrid strategies, such as heuristic algorithms, decomposition–reconstruction methods, and hybrid dynamics models.
- ML models typically have higher predictive accuracy than non-ML models.
- Despite the better performance of machine learning in COVID-19 prediction, it still has some limitations. Interpretability may limit the practical application of machine learning in infectious diseases.

This review provides systematic and in-depth theoretical support of AI for future researchers working in the field of infectious disease prediction, helping them to understand current technological advances and research directions more quickly. Secondly, it has been shown that ML has significant advantages in infectious disease prediction, especially hybrid modelling strategies, which show great potential in optimizing model accuracy. Finally, through these ML and hybrid modelling approaches, it provides deeper insights for future research, prompting researchers to explore more refined predictive models and drive technological innovation and development in public health.

## 5. Limitations and Future Challenges

Although we have systematically evaluated the application of machine learning in COVID-19 prediction, there are still key limitations and challenges that need to be addressed. The performance of ML models is highly dependent on the quality of the data. Additionally, in the field of public health, data across countries or regions may be inconsistent, missing or of poor quality. This limits the wide application of ML techniques in public health. Therefore, the integration of ML techniques into the practical operation of public health is a major challenge at present. Although modern ML and DL models have advantages in prediction accuracy, their “black box” nature makes it difficult for them to provide sufficient explanations, leading to a decrease in decision-makers’ trust in the models. Especially in the field of healthcare, decision-making errors can have serious consequences. Currently, researchers are trying to use methods such as Local Interpretable Model-agnostic Explanations (LIME), Shapley Additive Explanations (SHAP) to help explain the decision-making process of complex models, so as to improve the transparency and acceptance of public health decisions. In addition, some studies predicting COVID-19 have overlooked privacy risks in the use of health data, such as potential leaks of personal location or medical records, and lack in-depth discussions on the legality of data collection. Some models may have poor predictive performance for specific populations (such as ethnic minorities and residents in remote areas) due to data bias, leading to unfair allocation of public health resources. Current research focuses more on prediction accuracy and less on the impact of model applications on social ethics, such as the public trust crisis caused by excessive monitoring. These limitations reveal a deeper paradigmatic dilemma in the application of AI technology in public health-the conflict of values between technological rationality and health justice. Future research should consider balancing the relationship between technological efficiency and social ethics. For example, simulating extreme scenarios (such as climate refugee camp outbreaks) to test the robustness of algorithms, and embedding multidimensional fairness penalty terms in the loss function.

## Figures and Tables

**Table 1:** Major global infectious diseases and their hazards in the past 20 years.

Infectious Disease	Influence
SARS	In 2002–2003, over 8000 people were infected, resulting in approximately 800 deaths and a mortality rate of around 10%. Most cases are concentrated in China, Hong Kong, Taiwan, Canada, the United States, and other places.
H1N1	Over 1 billion people were infected, with an estimated death toll of 200,000 to 300,000, spreading globally.
MERS	Approximately 2500 people were infected and 900 people died, with a mortality rate of about 30%. It mainly spreads in the Middle East and also spreads to Asia, Europe, and the United States.
Ebola	Nearly 30,000 people were infected and approximately 11,000 people died. Mainly occurring in West Africa, the most severe outbreaks occurred in Liberia, Guinea, and Sierra Leone.
COVID-19	More than 700 million people have been infected and over 6 million have died (as of 2023), and the COVID-19 pandemic has rapidly spread to almost every country worldwide

**Table 2:** Research questions.

ID	Research Question
Q1	What type of data is used in the study?
Q2	How to handle incomplete, inaccurate, or noisy data?
Q3	Which ML methods are applied to COVID-19 trend prediction?
Q4	How to measure the prediction accuracy of ML technology?
Q5	What are the main challenges and limitations of ML in COVID-19 prediction?

**Table 3:** Strings used in the search.

Digital Databases	Search Query
Web of Science	(“Machine learning” OR “AI” OR “Deep learning”) AND (COVID-19) AND (“case” OR “trend” OR “outbreak” OR “transmissions” OR “Spread”) AND (“Prediction” OR “Forecasting”)
Elsevier	(“Machine learning” OR “AI” OR “Deep learning”) AND (COVID-19) AND (“case” OR “trend” OR “outbreak” OR “transmissions” OR “Spread”) AND (“Prediction” OR “Forecasting”)
Springer	(Machine learning OR AI OR Deep learning) AND COVID-19 AND (case OR trend OR outbreak OR transmissions OR Spread) AND (Prediction OR Forecasting)

**Table 4:** Assessment questions.

No.	Assessment Questions
AQ1	Are the aims of the research clearly defined?
AQ2	Is the topic of the article associated with the review?
AQ3	Are data sources provided in the article?
AQ4	Is the description of the data set clear in this article (data size, data splitting)?
AQ5	Are there any data preprocessing methods in the article?
AQ6	Are the research methods accurately described in the article?
AQ7	Did the study compare the proposed method with other methods?
AQ8	Is predictive performance measured and reported?
AQ9	Are the findings/results clearly reported?
AQ10	Are the limitations of research analyzed explicitly?

**Table 5:** Quality levels of selected studies.

Quality Level	*n*	%
Very high (17 ≤ score ≤ 20)	30	22
High (14 ≤ score ≤ 16)	65	48
Medium (11 ≤ score ≤ 13)	36	26
Low (0 ≤ score ≤ 10)	5	4
Total	136	100

**Table 6:** The main performance metrics used.

Metrics	Formula	*n*	%
RMSE	$\sqrt{\frac{1}{n_{samples}}\sum_{i=1}^{n_{samples}}{\left(y_{i}-{\hat{y}}_{i}\right)}^{2}}$	93	24.2
MAE	$\frac{1}{n_{samples}}\sum_{i=1}^{n_{samples}}\left|y_{i}-{\hat{y}}_{i}\right|$	62	16.1
MAPE	$\frac{1}{n_{samples}}\sum_{i=1}^{n_{samples}}\left|\frac{y_{i}-{\hat{y}}_{i}}{y_{i}}\right|$	50	13
R-square	$1-\frac{\sum_{i=1}^{n_{samples}}{\left(y_{i}-{\hat{y}}_{i}\right)}^{2}}{\sum_{i=1}^{n_{samples}}{\left(y_{i}-{\bar{y}}_{i}\right)}^{2}}$	47	12.2
MSE	$\frac{1}{n_{samples}}\sum_{i=1}^{n_{samples}}{\left(y_{i}-{\hat{y}}_{i}\right)}^{2}$	27	7
Accuracy	$\frac{TP+TN}{TP+TN+FP+FN}$	13	3.4
R	$\frac{Cov\left(x,y\right)}{\sqrt{var\left[x\right]var\left[y\right]}}$	6	1.6
Code openness	Y/N	23	16.9
Data Availability	Y/N	69	50.7
Others		79	22.5

**Table 7:** ML and non-ML methods used in the articles.

ML	Non-ML	Ref.	Best Model
RF, DT, KNR, Lasso, BR, KRR, Ransac Regressor, XGBoost, Elastic, Stacked LSTM, Stacked GRU	LR, Theilsen Regression, Holt Model	[21]	**RF, Prophet, Stacked LSTM**
Transformer-GCN, Transformer, LSTM, GRU	ARIMA, SARIMA	[23]	**Transformer-GCN**
XGboost, LSTM, NAIVEBAYESI	ARIMAI	[24]	ARIMAI
LASSO, LSTM, Interval type-2 fuzzy Kalman filter	ARIMA	[32]	**Interval type-2 fuzzy Kalman filter**
LSTM, Hybrid polynomial–Bayesian ridge regression model	ARIMA	[33]	**Hybrid polynomial–Bayesian ridge regression model**
Deterministic LSTM model, stochastic LSTM/MDN	LR	[34]	**LSTM**
LSTM	Google Cloud	[35]	**LSTM**
FNN, MLP, LSTM	ARIMA	[37]	**LSTM**
GAN-GRU, LSTM-CNN, RBM, GAN-DNN, CNN, LSTM, SVM	LR	[46]	**LSTM-CNN**
CEEMDAN-R-ILSTM-Elman	CEEMDAN-R-LSTM-ARIMA	[60]	**CEEMDAN-R-ILSTM-Elman**
SVR, MLP, RF	LR	[71]	LR
GRU, ColaGNN, CovidGNN, STAN-PC, STAN-Graph, STAN	SIR, SEIR	[72]	**STAN**
DT, RF, DL	ARIMA	[73]	**DT**
Naive method	Simple average, Moving average, Single exponential smoothing, Holt linear trend method, Holt–Winters method, ARIMA	[74]	**Naive method**
SVR, BR, RF, HW, XGBoost	ARMA, ARIMA, LR	[75]	ARIMA
XGBoost	ARIMAX	[76]	**XGBoost**
K-means, LSTM, Bi-LSTM	ARIMA, SMA-6, D-EXP-MA	[77]	**Bi-LSTM**
DTR, BeCaked, Ridge, SVR, LASSO, BR, RF	ARIMA	[78]	**BeCaked**
LSTM	Polynomial, VAR, LR, Sigmoid Curve models, Logistic model	[79]	**LSTM**
LSTM	ARIMA	[80]	**LSTM**
RF, SVR, LSTM, MTGP	LR	[81]	**MTGP**
RF, XGBoost	ARIMA, Prophet, GLMNet	[82]	ARIMA
RF	SMOreg, ARIMA, lBk, Gaussian Process, LR	[83]	ARIMA
SVM	AR, M5P, RSS	[84]	**SVM**
RF	KNN	[85]	**RF**
MLP, RBF, LSTM, ANFIS, GRNN	SEIRS	[86]	**ANFIS, RBF**
ANFIS	MLR	[87]	**ANFIS**
LSTM	ARIMA	[88]	**LSTM**
Ridge regression, ElasticNet, CGAN	Logistic, Lasso	[89]	**CGAN**
Ridge regression, Polynomial ridge regression, SVR	Polynomial regression, LR	[90]	**Polynomial ridge**
Fine Tree, Bagged Trees, Exponential GPR, Medium Tree, Boosted Trees, Trilayered Neural Network, Wide N.N., Matern 5/2 GPR, Squared exponential GPR, Rational Quadratic GPR	LR, Quadratic, Cubic, Inverse, ARIMA	[91]	**Fine tree**
AL-CNN	CAE	[92]	**AL-CNN**
FFNN	ETS, ARIMA	[93]	**FFNN**
LSTM, SLSTM	ARIMA, prophet	[94]	**SLSTM, LSTM**
GRU, LSTM	ARIMA, SARIMA	[95]	**LSTM, GRU**
ANNi	Gompertz model, Logistic, Bertalanffy model	[96]	**ANNi**
**Total number** (ML vs. Non-ML)	**31:5**		

## Data Availability

All data used in this study are public datasets that do not contain any personal privacy information and do not require ethical approval or permission.

## Abbreviations

The abbreviations employed in this manuscript are as follows:

AI	Artificial Intelligence
ML	Machine Learning
ARIMA	Auto Regressive Integrated Moving Average
SVM	Support Vector Machines
CNN	Convolutional Neural Networks
LSTM	Long Short-Term Memory Networks
WHO	World Health Organization
DL	Deep Learning
EHR	Electronic Health Records
MAPE	Mean Absolute Percentage Error
RMSE	Root Mean Square Error
MAE	Mean Absolute Error
MSE	Mean Squared Error
NN	Neural Networks
RF	Random Forest
GRU	Gated Recurrent
LR	Linear Regression
FNN	Feed Forward Neural Network
MLP	Multi-layer Perceptron
RNN	Recurrent Neural Networks
ICU	Intensive Care Units
DT	Decision Tree
GA	Genetic Algorithm
PSO	Particle Swarm Optimization
DE	Differential Evolution
FA	Firefly Algorithm
HS	Harmony Search
TLBO	Teaching–Learning-Based Optimization
BA	Bees Algorithm
mBA	mutation-based Bees Algorithm
LsOA	Lioness Optimization Algorithm
HBA	Honey Badger Algorithm
ABC	Artificial Bee Colony
CS	Cuckoo Search Algorithm
BBO	Biogeography-Based Optimization
IBAS	Improved Beetle Antennae Search Algorithm
SSA	Sparrow Search Algorithm
ANFIS	Adaptive Neuro-Fuzzy Inference System
GCN	Graph Convolutional Network
ANN	Artificial Neural Network
GEP	Gene Expression Programming
ELM	Extreme Learning Machine
LSSVR	Least Squares Support Vector Machine
WNN	Wavelet Neural Network
MARS	Multivariate Adaptive Regression Spline
LMBP	Levenberg–Marquardt Back Propagation
NAR	Nonlinear Auto Regressive
CEEMD	Complete Ensemble Empirical Mode Decomposition
VMD	Variational Mode Decomposition
AO-KELM	Adaptive Optimization-based Kernel Extreme Learning Machine
GLM	General Linear Model
NLP	Natural Language Processing
LIME	Local Interpretable Model-agnostic Explanations
SHAP	Shapley Additive Explanations

## Appendix A

**Table A1:** Quality assessment for each study.

No.	QA1	QA2	QA3	QA4	QA5	QA6	QA7	QA8	QA9	QA10	Total
1	2	2	1	0	2	2	2	2	2	0	15
2	2	2	1	2	2	2	2	2	2	1	18
3	2	2	2	2	1	2	0	1	1	0	13
4	2	2	1	2	2	2	2	2	2	0	17
5	2	2	2	1	1	2	2	2	2	2	18
6	2	2	2	2	2	2	2	2	2	0	18
7	2	2	1	1	2	2	2	2	2	1	17
8	2	2	0	1	0	2	2	2	2	0	13
9	2	2	1	1	1	2	2	2	2	0	15
10	2	2	2	2	2	2	2	2	2	0	18
11	2	2	2	0	2	2	0	2	2	0	14
12	2	2	1	1	2	2	2	2	2	2	18
13	2	2	1	2	2	2	2	2	2	1	18
14	2	2	2	2	1	2	2	2	2	0	17
15	2	2	0	2	2	2	2	2	2	0	16
16	2	2	1	2	2	2	2	2	1	0	16
17	2	2	2	0	0	0	2	2	2	0	12
18	2	2	1	0	1	2	0	1	1	0	10
19	2	2	1	2	0	2	2	2	2	0	15
20	2	2	2	2	0	2	2	2	2	0	16
21	2	2	2	2	2	2	2	2	2	0	18
22	2	2	1	0	2	2	2	2	2	0	15
23	2	2	2	2	0	2	2	1	2	0	15
24	2	2	2	2	2	2	2	2	2	0	18
25	1	2	1	1	0	2	2	2	2	2	15
26	2	2	1	0	1	2	2	2	2	0	14
27	2	2	2	2	1	2	2	2	2	2	19
28	2	2	0	2	0	2	2	2	2	0	14
29	2	2	2	2	2	2	2	2	2	0	18
30	2	2	1	2	0	2	2	2	2	0	15
31	2	2	0	2	0	2	2	2	2	0	14
32	2	2	2	2	2	2	2	2	2	0	18
33	2	2	2	2	1	2	2	2	2	0	17
34	2	1	1	1	1	1	2	2	2	0	13
35	2	2	0	0	2	2	0	2	2	0	12
36	2	2	1	1	0	2	2	2	2	0	14
37	2	2	1	2	1	2	2	2	2	2	18
38	2	2	1	1	2	2	2	2	1	0	15
39	2	2	1	2	2	2	2	2	2	2	19
40	2	2	0	0	0	2	2	2	2	0	12
41	2	2	2	1	0	2	2	2	2	0	15
42	2	2	2	2	0	2	2	2	2	0	16
43	2	2	2	2	0	2	0	2	1	0	13
44	2	2	1	1	0	2	2	2	2	0	14
45	2	2	1	2	0	2	2	1	1	0	13
46	2	2	1	2	0	2	2	2	2	0	15
47	2	2	2	2	1	2	2	2	2	0	17
48	2	2	1	2	1	2	2	2	2	0	16
49	2	2	1	2	0	2	2	2	2	0	15
50	2	2	1	0	2	2	2	2	2	0	15
51	2	1	2	0	0	2	2	2	2	0	13
52	2	1	1	2	2	2	2	2	2	2	18
53	2	1	1	2	0	2	0	2	1	0	11
54	2	2	1	2	0	2	1	2	2	0	14
55	2	2	1	0	0	2	2	2	1	0	12
56	2	1	2	0	0	2	2	2	2	0	13
57	2	1	2	1	0	2	2	2	2	2	16
58	2	2	2	0	0	2	2	2	2	0	14
59	2	2	1	0	0	2	2	2	2	0	13
60	2	2	0	1	0	2	2	2	1	0	12
61	2	2	2	0	0	2	2	2	2	2	16
62	2	2	2	2	0	2	2	2	2	0	16
63	2	2	0	1	0	2	2	2	2	0	13
64	2	2	1	0	1	2	2	2	2	0	14
65	2	2	2	0	0	0	2	2	2	0	12
66	2	2	2	1	1	2	2	1	1	0	14
67	2	2	2	0	0	2	2	2	2	0	14
68	2	2	1	0	0	2	0	2	2	0	11
69	2	2	2	0	0	2	2	2	2	0	14
70	2	2	1	0	0	2	0	2	2	0	11
71	2	2	0	0	0	2	2	2	2	0	12
72	2	2	1	1	1	2	0	2	2	2	15
73	2	2	1	0	0	2	2	2	2	0	13
74	2	2	1	1	0	2	2	2	2	0	14
75	2	2	2	2	0	2	2	1	1	0	14
76	2	2	2	1	0	2	2	2	2	0	15
77	1	1	1	1	2	2	0	1	1	2	12
78	2	1	0	1	0	2	2	2	1	0	11
79	2	1	2	0	0	2	2	2	2	0	13
80	2	2	2	2	2	2	2	2	1	0	17
81	2	2	2	2	0	2	0	2	1	0	13
82	2	2	1	0	0	2	2	2	2	0	13
83	2	2	1	1	2	2	2	2	1	0	15
84	2	2	2	2	2	2	0	2	1	2	17
85	2	1	1	0	0	2	0	2	2	0	10
86	2	2	2	2	0	2	2	2	2	0	16
87	2	2	2	1	0	2	0	2	1	0	12
88	2	2	1	1	0	2	2	2	2	2	16
89	2	2	1	1	0	2	2	2	2	0	14
90	2	2	2	1	0	2	2	2	2	0	15
91	2	2	0	2	2	2	2	2	2	0	16
92	2	2	1	2	0	2	2	2	2	1	16
93	2	2	1	0	0	2	0	2	2	0	11
94	2	2	2	1	2	2	2	2	2	0	17
95	2	2	2	2	0	2	2	2	2	2	18
96	2	2	1	1	0	2	2	2	1	0	13
97	2	1	1	1	0	2	0	2	2	0	11
98	2	2	1	2	0	2	0	2	1	0	12
99	2	2	2	1	2	2	2	2	1	0	16
100	2	2	1	1	2	2	0	2	2	1	15
101	2	2	1	2	0	2	2	2	2	0	15
102	2	2	2	2	1	2	2	2	2	0	17
103	2	2	1	2	2	2	2	2	2	2	19
104	2	2	2	2	0	2	0	1	1	2	14
105	2	2	2	2	0	2	2	2	2	0	16
106	2	1	0	0	0	2	2	1	1	0	9
107	2	2	1	2	0	2	2	2	2	0	15
108	2	2	1	2	1	2	2	2	2	0	16
109	2	1	1	0	0	2	2	1	1	0	10
110	2	2	0	1	0	2	2	2	2	0	13
111	2	2	2	1	2	2	0	2	1	0	14
112	2	2	1	1	0	2	0	1	1	0	10
113	2	2	0	2	0	2	2	2	2	0	14
114	2	2	1	2	2	2	2	2	2	0	17
115	2	2	2	2	2	2	2	2	2	0	18
116	2	2	2	2	2	2	2	2	1	0	17
117	2	2	2	2	0	2	2	2	2	0	16
118	2	2	1	2	0	2	2	2	1	0	14
119	2	2	1	1	0	2	2	2	2	2	16
120	2	2	2	1	0	2	2	2	2	0	15
121	2	2	0	2	0	2	2	1	1	0	12
122	2	1	1	0	0	2	2	2	2	0	12
123	2	2	1	2	2	2	2	2	2	0	17
124	2	2	2	2	0	2	2	2	2	0	16
125	2	2	2	2	0	2	2	2	1	0	15
126	2	2	2	1	2	2	2	2	1	0	16
127	2	2	1	1	2	2	2	2	1	0	15
128	2	2	1	2	0	2	0	2	1	0	12
129	2	2	1	2	0	2	2	2	1	0	14
130	2	2	1	2	1	2	2	2	2	1	17
131	2	2	1	1	0	2	2	2	1	0	13
132	2	2	1	0	2	2	2	2	2	0	15
133	2	2	1	2	0	2	2	2	2	0	15
134	2	2	1	2	0	2	2	2	2	0	15
135	2	2	1	0	0	2	2	2	2	0	13
136	2	2	1	2	0	2	2	2	1	0	14

**Table A2:** Main projects for data extraction.

Project	Attribute Name	Motivation
Publication characteristics	Title	Literature title
	Authors	The author of the document
	Keywords	Key words of literature
	Journal	Published journals
	Country	The country of study
	Year	Year of publication
Data	Data sources	Where did the data information come from?
	Number of samples	The total number of samples in the dataset
	Input features	Variables in data sets used to train ML models
	Target variable	Variables that the model tries to predict or explain
	Feature Selection	Select the most relevant feature from all available features
	Data Correction	The process of cleaning and transforming the original data
	Data Split	Divide the data set into training set and test set according to a certain proportion
Methods	Models	ML methods used in literature
	Intelligence Algorithms	Intelligent optimization algorithm used in literature
Performance	Index	Evaluation index of model performance used in the literature
	Best model	The model with the best prediction performance
